# A Study of Differential Gene Expression and Core Canonical Pathways Involved in Rhenium Ligand Treated Epithelial Mesenchymal Transition (EMT) Induced A549 Lung Cancer Cell Lines by INGENUITY Software System

**DOI:** 10.4236/cmb.2022.121002

**Published:** 2022-03-07

**Authors:** Christopher Krauss, Chelsey Aurelus, Kayla Johnston, Joseph Hedley, Satyendra Banerjee, Sarah Wisniewski, Quentin Reaves, Khadimou Dia, Shenell Brown, Victoria Bartlet, Sheritta Gavin, Jazmine Cuffee, Narendra Banerjee, Kuldeep Rawat, Santosh Mandal, Zahidur Abedin, Somiranjan Ghosh, Hirendra Banerjee

**Affiliations:** 1 Department of Natural Sciences and Department of Health and Human Studies, Elizabeth City State University, University of NC, Elizabeth City, NC, USA; 2 Department of Chemistry, Morgan State University, Baltimore, MD, USA; 3 PrimBio Research Institute LLC, Exton, PA, USA; 4 Department of Pediatrics and Child Health, Howard University Medical School, Washington DC, USA

**Keywords:** Rhenium Compounds, Lung Cancer, Epithelial

## Abstract

Rhenium compounds have shown anti-cancer properties against many different types of cancer cell lines; however, the cellular signaling mechanisms involved in the cytotoxic properties of rhenium-based compounds were never deciphered or reported. In this manuscript, we report the results of an investigation done by RNA sequencing of rhenium treated A549 lung cancer cell lines along with an untreated vehicular control, analyzed by the Ingenuity Pathway Analysis (IPA) software system to decipher the core canonical pathways involved in rhenium induced cancer cell death. A549 EMT lung cancer cell lines were treated with rhenium ligand (Tricarbonylperrhenato(bathocuproine)rhenium(I), PR7) for seven days along with vehicular control. RNA was isolated from the treated and control cells and sequenced by a commercial company (PrimBio Corporation). The RNA sequencing data was analyzed by the INGNUITY software system and the core canonical pathways involved with differential gene expression were identified. Our report is showing that there are several cellular pathways involved in inducing cell death by rhenium-based compound PR7.

## 1. Introduction

Rhenium based compounds have remained an interesting therapeutic strategy in the cancer biology field for decades. Rhenium ligands were reported to be toxic to cancerous cells while respiting the healthy cells; thus rhenium could potentially be a very effective drug for cancer treatments [1]. In this study, we tested the cytotoxicity of a rhenium ligand, Tricarbonylperrhenato(bathocuproine)rhenium(I) (PR7), against a GFP labeled vimentin gene knock in by CRISPR modified EMT model A549 lung cancer cell lines. These cell lines were created at ATCC (USA) to study therapeutic efficacy of anti-cancer compounds to prevent epithelial mesenchymal transition (EMT).

PR7 was previously reported to be bioactive against endometrial cancer cell lines [2]. In this study, we treated the vimentin knock in A549 lung cancer cell lines with PR7 after inducing EMT by TGF beta treatment and determined the cytotoxic effects of the drug by MTT assay technique. RNA was isolated from the PR7 treated EMT induced A549 cancer cells along with vehicular control (DMSO treated). The RNA was sequenced at a commercial RNA sequencing laboratory (PrimBio, LLC, USA). This sequencing data was then analyzed by the INGENUITY (IPA) software licensed from Qiagen Corporation, USA, to identify the potential effects of PR7 on gene expression and cellular signaling. In this manuscript, we report four core cellular canonical pathways that IPA reported and deciphered the differential gene expression due to the rhenium ligand treatment.

## 2. Materials and Methods

The rhenium ligand PR7 was synthesized as described previously [2]. The drug was dissolved in DMSO to form a solution.

The A549 EMT cell line was purchased from ATCC, USA and cultured in F-12K medium supplemented with FBS, Penicillin, and Streptomycin. The cells were kept in a 37°C incubator with 5% CO_2_.

The MTT assay reagents were purchased from R&D Systems, USA and the experiment was performed following the manufacturer’s protocol after exposure to 1 μM of PR7 for 48 hours. The results were read in a standard plate reader.

EMT was induced in the A549 cells by treating with 2.5 ng/ml TGF-ß for seven days along with 1 μM PR7 and TGF-ß with 1 μM DMSO treated cells only as vehicular control for the same time period.

RNA was isolated from TGF-ß and PR7 treated A549 cells and vehicular control cells after seven days treatment by using a total RNA isolation kit from Signosis Corporation, USA and then sent to PrimBio Corporation, USA for RNA sequencing. The data generated was then analyzed by the INGENUITY SYSTEM (IPA) software licensed from Qiagen Corporation, USA.

## 3. Results

**Figure 1.**
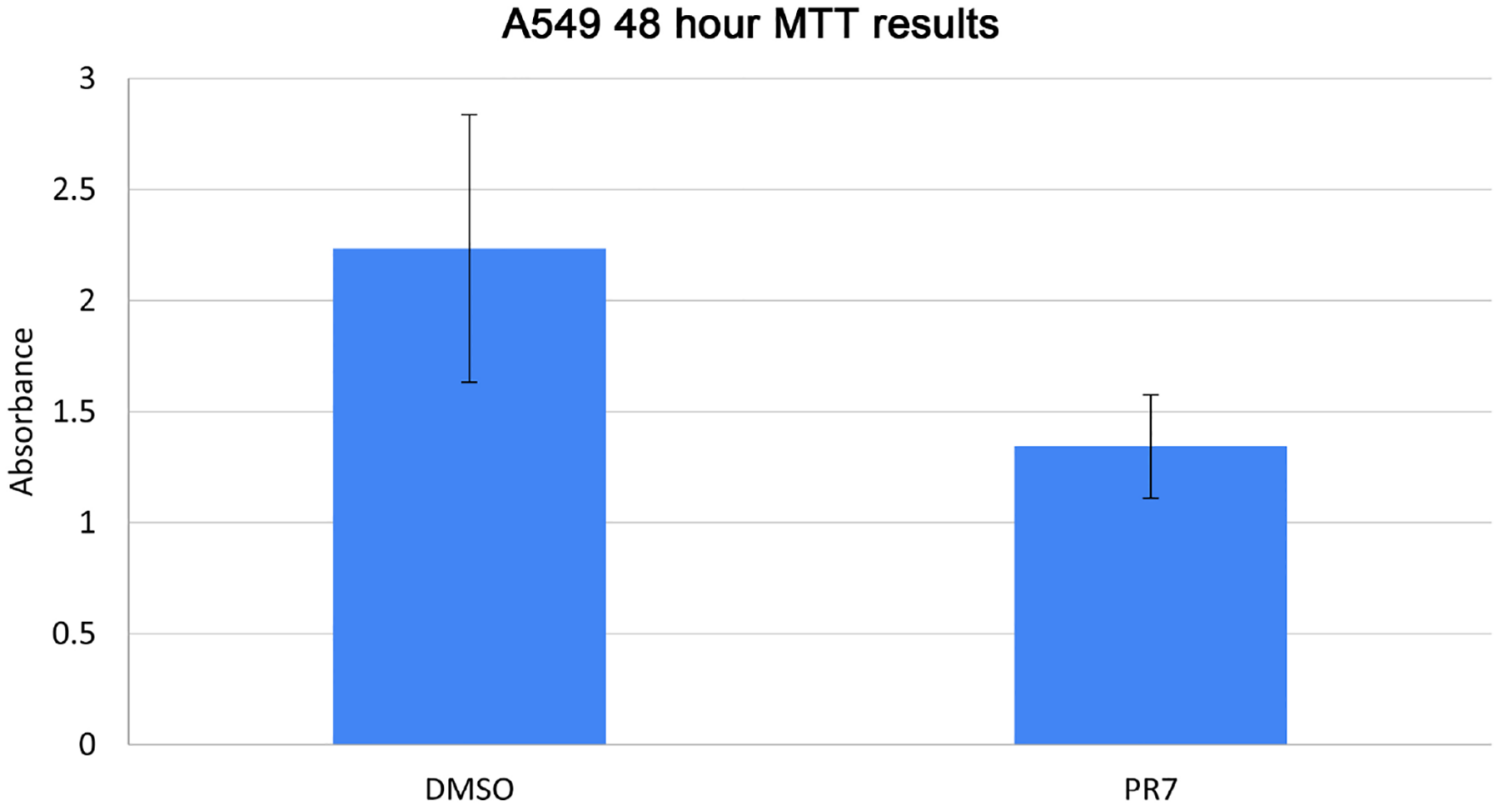
48 hours MTT results of DMSO control vs 1 μM PR7 treated cells. A549 EMT Cells were grown in a 5% CO_2_ incubator until confluent then treated with TGF beta and 1 μM of PR7 or an equal concentration of TGF beta and DMSO and returned to the incubator for 48 hours. Then MTT assay was performed, results showing increased cell death in PR7 treated cells.

**Figure 2.**
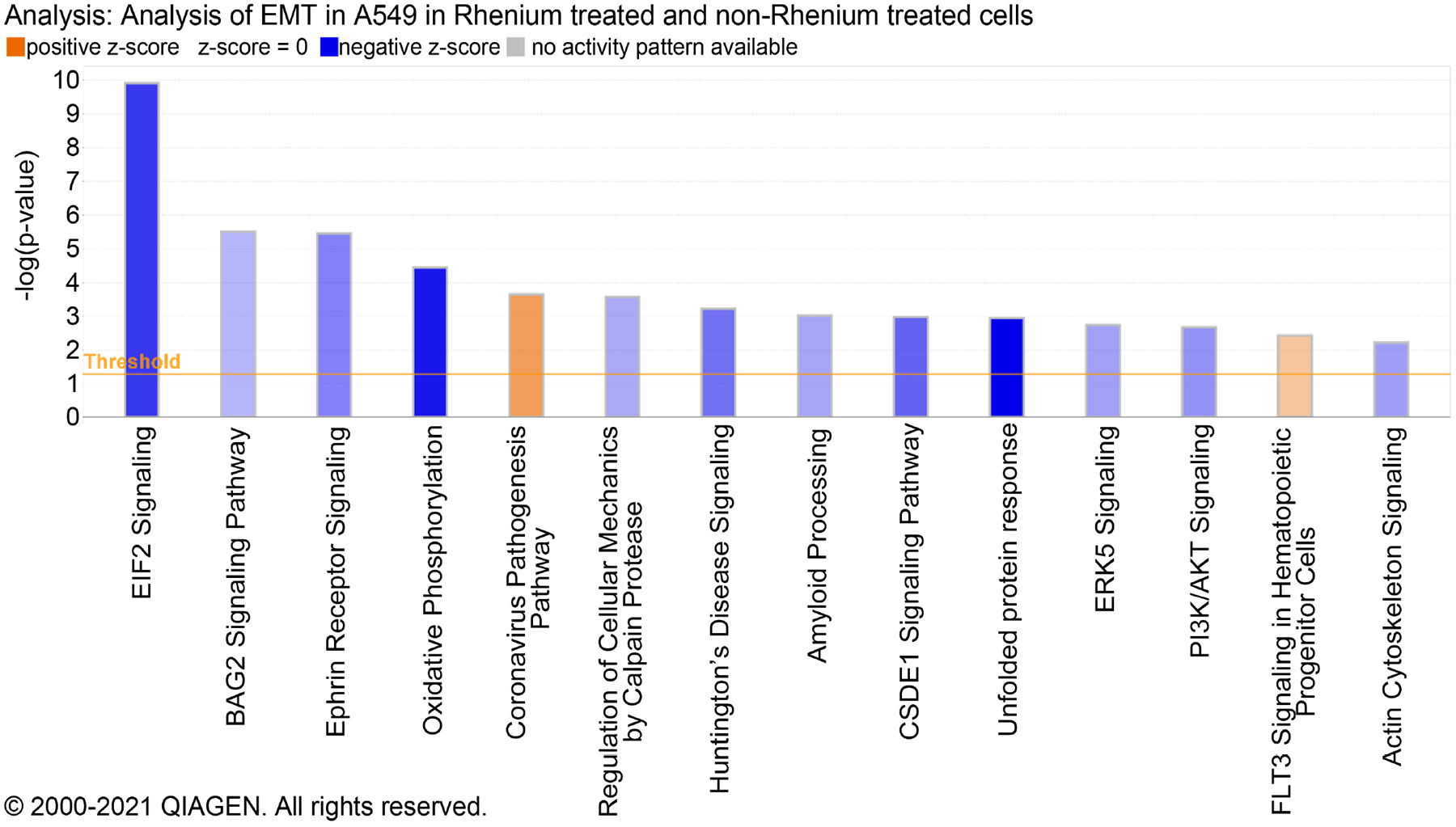
Core canonical pathways induced by PR7. This figure displays the core canonical pathways that PR7 treatment is likely to alter. Pathways shown in blue are anticipated to be downregulated, while an orange display shows the pathway to be likely upregulated.

Performing a MTT assay on A549 cells exposed to 1 μM PR7 showed an increase in cell death compared to cells exposed to DMSO alone as a vehicular control (Figure 1). This proved that PR7 is bioactive in the A549 EMT cell line. We then analyzed by RNA sequencing the differential gene expression and by IPA analysis the core canonical pathways involved in PR7 treatment. Table 1 shows the major differentially expressed genes in response to PR7 treatment. Figure 2 shows the different signaling pathways involved as deciphered by the IPA system due to PR7 treatment, in order to decipher the differential gene expression in A549 cells that were exposed to PR7, RNA isolated and sent for sequencing. There were two groups of A549 cells RNA that were sent for sequencing, both group of cells were exposed to TGF-ß to induce EMT, but only one group was exposed to PR7. The data generated from this sequencing was uploaded into the IPA software for a comparison analysis. This showed the differences in gene expression in the EMT induced A549 cells due to treatment with PR7. The data showed differences in over 90 core canonical pathways and calculated the changes in 9457 genes. Naturally all these pathways and genes were not relevant to lung cancer, so we selected four canonical pathways to examine and sorted the software’s data to find the significant down and upregulated genes that could be associated with lung cancer.

## 4. Discussions

Our study showed that the rhenium ligand PR7 is cytotoxic to the A549 cancer cell line and induces differential gene expression. The drug treated CRISPR Cas9 modified vimentin-GFP knock in A549 lung cancer cell lines showed several downregulated and upregulated genes that are involved in cancer biogenesis pathways. We reported several such upregulated and downregulated genes relevant to lung cancer in Table 1 while Figure 2 shows the important core canonical pathways involved in PR7 treatment. Analyzing the downregulated genes, PR7 decreased expression of these oncogenes that helps in cancer progression. One of the genes found to be downregulated, FLNA, was reported to decrease drug resistance of lung cancer cells to gefitinib [3]. Gefitinib works by targeting the gene EGFR. This gene was also found to be downregulated due to the PR7 exposure, though not to the same degree as the other reported genes. Downregulation of EGFR was by an expression fold change of −3.334 due to PR7 exposure. This suggests that while PR7 does appear to show promising results on its own to treat lung cancer, it might also be able to be combined with gefitinib to increase efficacy. However, this combination was not able to be tested in our lab at this time, so the combinations efficacy cannot be stated certainly. Analyzing the upregulated genes, LARP4 upregulation was the only upregulated gene that was detrimental to cancer as its upregulation can inhibit migration and invasion [4]. The relevancy of the differential expression of these up and down regulated genes should be further investigated.

A brief discussion of the core canonical pathways that were identified by the IPA software system is described as follows.

The BAG2 signaling pathway was downregulated due to treatment with the PR7. This could delay tumor development due to the P53 downregulation at the end of the pathway. While P53 is a tumor suppressor gene, it is the most commonly mutated gene in cancers [5]. This mutation can occur due to BAG2 promoting the accumulation of mutated P53 [5]. When examining the molecules function of EIF2 signaling, which was the pathway with the best p-value, this pathway has some publications linking it to cancer. Inhibiting the EIF2 signaling pathway has been linked to a reduction in tumor growth in gastric cancers [6]. One study identified EIF2ß as a potential therapeutic target for non-small cell lung cancers [7]. Looking into the EIF2 signaling pathway more thoroughly in IPA showed that in the treated A549 cells, EIF2ß was downregulated.

Oxidative phosphorylation is typically shifted away from in cancers with most cancers favoring glycolysis as per the Warburg effect [8]. However, studies are showing that lung cancers do require oxidative phosphorylation to develop [8] [9]. Lung cancer cells with SMARCA4 mutations appear to be sensitive to inhibiting oxidative phosphorylation [9].

The ephrin signaling pathway is typically overexpressed in a variety of tumors. This promotes tumorigenesis, metastasis, and cancer stem cell regeneration [10]. Studies have identified this pathway as a target for drug development. PR7 was predicted to inhibit this pathway. A possible reason for this inhibition could be the downregulation of SHC. SHC binds with EPHA2, which is known to regulate tumor growth, migration, and invasiveness [11] [12]. However, in this same pathway, STAT3 was found to be upregulated which can promote metastasis [13]. Thus, our study shows the application of computational analysis by the IPA Software of molecular data obtained from RNA Sequencing and deciphering along with differential gene expression studies, analysis of the cellular core canonical pathways involved in potential anticancer therapeutic properties of the novel Rhenium ligand PR7.

## Figures and Tables

**Table 1.** Differentially expressed genes due to PR7 treatment with known role in lung cancer.

Gene name	Expression Fold Change	Effect on lung cancer
ACTG1	−66.515	Found to be upregulated in small cell lung cancers. Overexpression has been linked to higher metastatic potential in hepatocellular carcinoma. This suggests that ACTG1 is likely assisting in cancer metastasis in lung cancers [14].
ENO1	−177.945	Found to induce tumor growth and metastasis in vivo in lung adenocarcinomas [15]
FLNA	−67.865	High expression induces resistance to gefitinib, while lowering expression restores sensitivity to gefitinib. Lower expression is also able to induce apoptosis [3].
LARP4	25.148	Typically has a lower expression in non-small cell lung cancers including A549. Higher expression could inhibit migration and invasion [4].
RPL19	−115.264	Lowering RPL19 levels was found to inhibit the growth of lung cancers that typically have an overexpression of RPL19. It is a proposed target for immunotherapy [16].
RPS16	−73.909	Higher levels linked to lower survival rate in lung cancer patients [17].
RPS27A	−88.972	Direct transcriptional target of p53 that is highly overexpressed in lung cancer. Appears to be a promising target for treatment [18].
TM4SF1	−86.345	Upregulated in non-small cell lung cancers. Promotes cell proliferation, migration, and invasion while also inhibiting apoptosis [19].
UBB	−84.321	Overexpression leads to a lower survival rate in lung cancer patients [20].
YBX1	−75.424	High levels have been associated with poor prognosis in cancer patients [21].
